# Health-related quality of life of antiretroviral treatment defaulters in Botswana

**DOI:** 10.4102/sajhivmed.v17i1.475

**Published:** 2016-10-31

**Authors:** Nnamdi O. Ndubuka, Hyun J. Lim, Dirk M. van der Wal, Valerie J. Ehlers

**Affiliations:** 1 Department of Health Studies, University of South Africa, South Africa; 2 Northern Inter-Tribal Health Authority, Prince Albert, South Africa; 3 Department of Community Health and Epidemiology, College of Medicine, University of Saskatchewan, Canada

## Abstract

**Background:**

Antiretroviral therapy (ART) improves patients’ health-related quality of life (HRQoL). Defaulting from ART has detrimental consequences, including the development of viral resistance, treatment failure and increased risks of disease progression. Little is known about the quality of life of ART defaulters and reasons for discontinuing their ART.

**Objectives:**

This study sought to measure the HRQoL of ART patients in Botswana who were on ART for up to 5 years but had discontinued treatment for at least 3 months, and to identify factors associated with ART defaulting.

**Method:**

We conducted a cross-sectional study with 104 eligible respondents in four ART clinics in south eastern Botswana. We assessed respondents’ HRQoL using the World Health Organization Quality of Life Questionnaire for HIV short form. Clinical information was obtained from respondents’ medical records. Data were analysed using SAS version 9.2.

**Results:**

Reasons for discontinuing ART were inaccessible clinics (22.4%), feeling better (21.4%), running out of pills (11.2%), depression (8.2%), lack of care and/or support (8.2%), failure to understand instructions (7.7%), medications’ side effects (6.1%) and alcohol abuse (3.1%). In multivariate analyses, respondents aged 36–45 years had a 0.03 lower independence HRQoL score compared to those aged 35 and younger (β = -0.03; 95% confidence interval: -1.72, -1.66). Despite defaulting from their ART, respondents’ calculated HRQoL scores were moderate.

**Conclusion:**

This study highlights the need to enhance ART adherence in order to improve the HRQoL of people living with HIV and/or AIDS.

## Introduction

### Background

Human immunodeficiency virus (HIV) is no longer a fatal disease because antiretroviral therapy (ART) has become available. ART reduces HIV-related morbidity and mortality and suppresses viral replication, thus increasing the individual’s CD4 counts and decreasing their viral load. Botswana was the first country in Africa to provide ART to people living with HIV and/or AIDS (PLWHA) through a national programme. According to the United Nations Joint Programme on HIV and/or AIDS,^1^ new HIV infections in Botswana decreased from 15 000 in 2005 to 9100 in 2013 and AIDS-related deaths decreased from 14 000 in 2005 to 5800 in 2013, and an estimated 213 953 out of a possible 320 000 PLWHA received ART in Botswana.^1^

The World Health Organization (WHO)^2^ defined quality of life (QoL) as ‘… an individual’s perception of his/her position within the context of culture and value systems in which he/she lives in relation to the individual’s goals, expectations, standards, and concerns’. QoL could also be regarded as a person’s satisfaction and happiness with his or her daily life.^3^ Health-related quality of life (HRQoL) is an important aspect that determines the level of well-being of PLWHA because ART adds years to these individual’s lives. ART improves the HRQoL of PLWHA, according to recorded clinical evidence.^4^ However, ART adherence is essential to derive maximum benefits and an improved HRQoL over time.^5,6,7,8^ Although there are other definitions of HRQoL, those provided by WHO were accepted for the purpose of this study.

Non-adherence to the prescribed ART regimen has serious consequences for PLWHA, for their communities and for healthcare services. Consequences of ART non-adherence include the development of HIV resistance to antiretrovirals (ARVs), necessitating the use of expensive and complicated second- or third-line ART regimens with increased chances of developing side effects. Other consequences of ART non-adherence involve the risk of disease progression to AIDS, developing opportunistic infections, decreased HRQoL, increased chances of infecting other people with HIV, and death.^9,10,11,12^ Consequently, ART services should identify defaulters and their reasons for discontinuing ART so as to institute timely interventions to retain patients on ART regimens and to improve their HRQoL.

Previous studies addressed the HRQoL of PLWHA in Botswana, but limited information is available about the HRQoL of ART defaulters as the latter group comprises a largely inaccessible population. This study therefore attempted to measure the HRQoL of Botswana patients on ART for at least 5 years who had defaulted ART for at least 3 months and to identify reasons why these patients discontinued their ART.

## Methods

### Study setting, population and sample

Data collection was conducted at four ART clinics in four different health districts in south eastern Botswana. The study population comprised adult ART patients (aged 21 or older) who had commenced ART from January 2002 to December 2007, and who had stopped taking ARVs for at least 3 months. ART patients younger than 21 years of age or who had defaulted their ART for less than 3 months were excluded. From the four participating ART clinics’ registers, 362 adult patients who had been on ART for at least 5 years and who had discontinued their treatment for at least 3 months were identified. Of these 362 ART defaulters, 128 could be approached, but 18.8% (*f* = 24) refused to participate in the study. Thus, a total of 104 eligible respondents participated in the study across four study sites between 01 September and 15 November 2012. The response rate was therefore 81.2% (*n* = 104).

## Study design

This was a cross-sectional, quantitative and exploratory study to describe ART defaulters’ HRQoL at one point in time when data collection for this study took place.

### Measures and instrument

Three trained research assistants read the items to the ART defaulters and recorded their responses on the questionnaires. This was done due to concerns regarding potential low literacy rates. The instrument comprised a 45-item questionnaire, designed by the researchers, and the WHO Quality of Life Questionnaire for HIV short form (WHOQoL-HIV BREF). A bilingual expert translated both instruments into the local language *Setswana*, and then back translated these into English to ensure that the meaning of the English and Setswana items corresponded.

Section A of the questionnaire requested information about ART patients’ socio-demographic characteristics including age, gender, marital status, number of children, educational level, religion, employment status and HIV status disclosure. Clinical aspects such as the most recent CD4 cell count and viral load, the year when ART commenced, the year of HIV diagnosis, duration on ART, reasons for discontinuing ART and opportunistic infections were obtained from respondents’ medical records. Cronbach’s alpha coefficient for the questionnaire was 0.75, indicating an acceptable level of internal consistency.

The WHOQoL-HIV BREF instrument is a short version of the WHOQoL-100 instrument developed by the WHO HIV and/or AIDS Quality of Life Group for cross-cultural applications. It has 31 items representing six domains: physical (4 items), psychological (5 items), level of independence (4 items), social relationships (4 items), environment (8 items), spirituality (4 items) and two additional items (overall HRQoL and general health).^13,14^ It is available in more than 20 languages and can be used in different settings, but ‘… the results are comparable across different cultural settings’.^2^ All the individual items are rated on a 5-point Likert scale, where ‘1’ indicates the lowest level of feelings and ‘5’ indicates the highest level of feelings. This instrument asks questions about ‘how satisfied, how much, how completely, how bothered’ ART defaulters felt about different aspects of their lives during 2 weeks preceding data collection. For example, in response to the question ‘How satisfied are you with your capacity to work?’, 1 would indicate ‘very dissatisfied’ and 5 would indicate ‘very satisfied’. These responses to individual aspects within the six domains are reported in terms of mean scores, with 5 being the maximum score.

In this study, the calculated domain and overall QoL scores were based on the WHOQoL-HIV BREF scoring and coding methodology. The mean scores of all items in each domain were multiplied by four in order to make domain scores directly comparable with the scores used in the WHOQoL-100. The domain scores therefore ranged from the lowest count of four to the highest count of 20. The maximum total HRQoL score for the six domains combined was therefore 120.

Three research assistants, fluent in both English and Setswana and trained as HIV counsellors, were recruited and trained by the first author. They asked every person the same questions in the same order in his or her preferred language (English or Setswana) and completed the questionnaire on behalf of the ART defaulter. The questionnaire was pretested on three eligible ART defaulters who met the inclusion criteria. These three persons encountered no problems in answering the questions, and the research assistants encountered no challenges in recording the answers on the questionnaires. The results of these three completed questionnaires were excluded from the data analysis of the actual study.

#### Ethical considerations

The research proposal was approved by Botswana’s Ministry of Health with reference number PPME-13/18/1 Vol VII (318). Managers of the four participating ART clinics also approved the proposal and granted permission for data collection at their clinics. The Higher Degrees Committee of the Department of Health Studies, University of South Africa, granted ethical approval for the study with reference number HSHDC/7/2012. Every ART defaulter provided informed written consent. Refusal to participate in the study did not affect their treatment in any manner whatsoever. Unique code numbers were used instead of respondents’ names to ensure confidentiality and anonymity of respondents. All completed questionnaires were securely locked up. All information was entered into a password-protected computer to which only the researchers had access. The completed questionnaires and the computer records will be destroyed after acceptance of the research report.

#### Statistical analysis

Descriptive statistics were used to describe the demographics and health characteristics of the ART defaulters with means, ranges, frequency distributions, percentages and standard deviations. Mean estimates with standard deviations of the HRQoL outcome domains (physical, psychological, level of independence, social relationships, environment and spirituality) were reported. Spearman’s correlation coefficients were used to identify the relationship between dependent and independent variables. To understand the factors associated with HRQoL among ART defaulters, a series of bivariate associations were performed, selecting key variables related to respondents’ demographic and clinical characteristics such as their coping experiences with life’s challenges, ART adherence levels and healthcare system-related issues. All the independent covariates were initially tested for potential relationships with each HRQoL outcome and with the total QoL. All significant independent covariates at univariate regression analyses were examined and only significant covariates were included in subsequent multivariate regression models. Also stepwise forward model building strategies were used. Interactions between variables in the multivariate models were examined.

Statistical significance was set at *p* < 0.05. All statistical analyses were performed using the SAS version 9.2 programme.^15^

## Results

### Socio-demographic and clinical characteristics of the study population

Of 128 contacted ART defaulters, 104 eligible ART defaulters participated in the study, resulting in a response rate of 81.2%. (Participation was voluntary and no reasons were provided by the 24 ART defaulters for refusing to participate.) Of the 104 respondents, 51 (49.0%) were females, 53 (51.0%) were males, the mean age was 37.9 years and the standard deviation (SD) was 11. Of the respondents, only 4 (3.9%) were married, 29 (28.2%) had completed primary education and 5 (4.9%) had completed college or vocational training. Regarding restarting ART, 53.5% of respondents who defaulted had restarted their ART at the time of data collection.

The respondents’ reasons for discontinuing ART included inaccessible clinics (22.4%), feeling better (21.4%), running out of pills (11.2%), depression (8.2%), lack of care and/or support (8.2%), failure to understand instructions (7.7%), medications’ side effects (6.1%) and alcohol abuse (3.1%).

Of the respondents, 93.1% correctly understood that ART adherence implies taking ARVs at the right time and the right doses as prescribed, while 6.9% lacked adequate knowledge about ART adherence. Other characteristics of respondents are summarised in Table 1.^16^

**TABLE 1:** Socio-demographic and disease-related characteristics of respondents (*n* = 104).

Characteristic	Variable	*n*	%
Age	15–25	16	15.4
	26–35	25	24.0
	36–45	37	35.6
	46–55	21	20.2
	56 or older	5	4.8
Gender	Male	53	51.0
	Female	51	49.0
Marital status	Single	76	73.1
	Married	4	3.9
	Cohabitating	12	11.5
	Widowed	5	4.8
	Divorced	0	0.0
	Separated	7	6.7
Education	None	33	32.0
	Primary school	29	28.2
	Junior secondary	29	28.2
	Junior secondary with additional training	6	5.8
	Senior secondary	1	1.0
	College or vocational training	5	4.9
	University	0	0.0
Religion	Christian	356	78.1
	Islam	4	0.9
	Traditional	61	13.4
	Others	35	7.7
Employment	Employed	36	35.3
	Unemployed	54	52.9
	Self-employed	5	4.9
	Volunteer	2	2.0
	Student	5	4.9
Monthly income (BWP)	0–999	76	74.5
	1000–1999	11	10.8
	2000–2999	7	6.9
	3000 and more	8	7.8
Duration on ART before defaulting	3–6 months	33	33.7
	7–11 months	6	6.1
	1–2 years	29	29.6
	3–4 years	14	14.3
	5 years and more	16	16.3
Duration without ART (default period)	3–6 months	34	35.0
	7–11 months	6	6.2
	1–2 years	43	44.0
	3–4 years	11	11.3
	5 years and more	3	3.1
Restarted ART by time of interview	Yes	53	53.5
	No	46	46.5

ART, antiretroviral therapy; BWP, Botswana Pula.

*Source*: Ndubuka NO. Quality of life of ART patients in Botswana: Coping with challenges [unpublished D Lit et Phil thesis]. Pretoria: University of South Africa; 2015.

With regard to ART defaulters’ coping experiences, only 17.8% had previous experiences with overcoming difficulties other than being HIV-positive. Most of the respondents (87.3%) did not belong to any community-based support groups.

### Health-related quality of life scores of respondents

**FIGURE 1:**
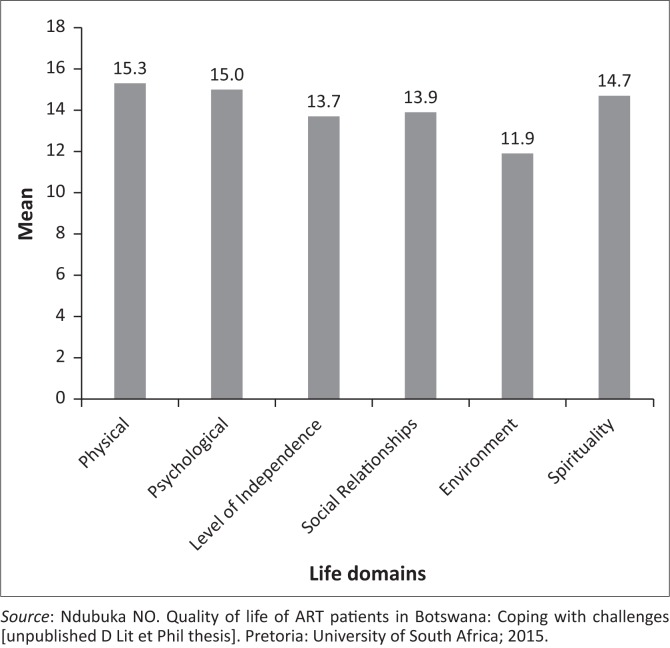
Mean quality of life domains (*n* = 104).

Figure 1 presents the HRQoL mean scores across the various QoL dimensions (with a maximum score of 20 in each dimension) among respondents. The higher HRQoL scores were observed in the physical (mean = 15.3, standard deviation [SD] = 3.2), psychological (mean = 15.0, SD = 2.8) and spirituality (mean = 14.7, SD = 2.2) domains, while lower scores were observed in level of independence (mean = 13.7, SD = 4.1), social relationships (mean = 13.9, SD = 3.7) and environment (mean = 11.9, SD = 3.4) domains. The overall mean HRQoL score of respondents (across all domains with a maximum score of 120) was relatively high (mean = 84.6, SD = 14.8).

### Factors associated with HRQoL scores of respondents

Table 2 shows the association between respondents’ characteristics and HRQoL domain scores. Multivariate analyses indicate that respondents’ ages, incomes, coping experiences and the number of people disclosed about their HIV-positive status were significant variables in the independence HRQoL domain. Respondents aged 45 years and older had a 2.27 lower independence HRQoL score compared to those aged 35 and younger (β = -2.27; 95% confidence interval (CI): -4.18, -0.36).

**TABLE 2:** Factors associated with HRQoL of respondents (*n* = 104).

Variables	Findings	Physical		Psychological		Level of independence		Social relationship		Environment		Spirituality	
		*n*	95% CI	*n*	95% CI	*n*	95% CI	*n*	95% CI	*n*	95% CI	*n*	95% CI
Intercept	-	14.2	13.5, 14.9	14.1	13.4, 14.8	14.6	13.4, 15.9	12.3	11.4, 13.2	11.5	10.8, 12.1	14.8	14.2, 15.5
Age	<36	-	-	-	-	ref	-	-	-	-	-	-	-
	36–45	-	-	-	-	-0.03	-1.72, 1.66	-	-	-	-	-	-
	>45	-	-	-	-	-2.27	-4.18, -0.36	-	-	-	-	-	-
Education	Yes	-	-	ref	-	-	-	ref	-	ref	-	-	-
	None	-	-	-1.53	-2.61, -0.44	-	-	-1.35	-2.69, -0.02	-2.79	-4.07, -1.51	-	-
Income	≤2000	-	-	-	-	ref	-	-	-	-	-	-	-
	>2000	-	-	-	-	3.56	1.44, 5.69	-	-	-	-		-
Employment	Yes	ref	-	-	-	-	-	ref	-	-	-	-	-
	No	-1.37	-2.51, -0.23	-	-	-	-	-1.34	-2.59, -0.09	-	-	-	-
Coping experience	No	ref	-	ref	-	ref	-	ref	-	-	-	-	-
	Yes	-3.83	-5.33, -2.34	-2.41	-3.72, -1.11	-2.39	-4.31, -0.47	-2.91	-4.53, -1.27	-	-	-	-
Number of people disclosed	≤10	-	-	-	-	ref	-	-	-	-	-	-	-
	>10	-	-	-	-	1.95	0.31, 3.59	-	-	-	-	-	-
Number of people taking ART at home	None	-	-	-	-	-	-	ref	-	-	-	ref	-
	One or more	-	-	-	-	-	-	2.27	0.84, 3.71		-	1.45	0.45, 2.45
Reason for missing ARV	Felt better	-	-	-	-	-	-	-	-	-	-	-	-
	Forgot	-	-	-	-	-	-	-	-	-	-	0.07	-1.86, 1.99
	Depressed	-	-	-	-	-	-	-	-	-	-	-1.10	-2.86, 0.67
	Others	-	-	-	-	-	-	-	-	-	-	-1.17	-2.08, -0.26

ART, antiretroviral therapy; ARV, antiretroviral; CI, confidence interval; HRQoL, health-related quality of life; ref, reference group.

The respondents’ mean education level was significantly associated with psychological (β = -1.53; 95% CI: -2.61, -0.44), social relationship (β = -1.35; 95% CI: -2.69, -0.02) and environmental (β = -2.79; 95% CI: -4.07, -1.51) HRQoL mean scores, but not with the other scores. Respondents’ employment status was associated with their mean scores in the physical (β = -1.37; 95% CI: -2.51, -0.23) and social relationship HRQoL domain (β = -1.34; 95% CI: -2.59, -0.09). In relation to the psychological domain, only education and previous coping experiences were associated with psychological HRQoL mean scores. The psychological HRQoL mean scores of respondents with no education were 1.53 points lower compared to those who had some education, after controlling for previous coping experiences (95% CI: -2.61, -0.44). The psychological HRQoL mean scores of respondents with previous coping experience were 2.41 points lower compared to those with no coping experiences (95% CI: -3.72, -1.11).

Regarding the level of independence domain, respondents whose monthly incomes exceeded BWP2000 showed 3.56 higher independence HRQoL mean scores compared to those with monthly incomes of less than BWP2000 (95% CI: 1.44, 5.69) after controlling for other significant factors (age, previous coping experience and the number of persons to whom respondents had disclosed their HIV-positive status). Respondents with previous coping experiences had a 2.39 lower independence HRQoL mean score compared to those with no coping experiences, after controlling for the other significant factors (95% CI: -4.31, -0.47). Concerning the environmental domain, the multivariate regression analysis showed that only education was associated with the environmental HRQoL mean score. Respondents who had no education had a 2.79 lower environmental HRQoL mean score compared to those with some education (95% CI: -4.07, -1.51). In the spirituality domain, respondents who had other people taking ART in their homes showed a 1.45 higher spirituality mean score than those who did not have other persons taking ARVs in their homes (95% CI: 0.45, -2.45) (Table 2).

## Discussion

Despite defaulting from their ART, respondents in this study obtained moderate HRQoL mean scores. The low HRQoL mean score findings in the social and environmental domains might signify inadequate social support structures, stigma, poor financial resources, poor physical living conditions and insecurity.

ART defaulters in this study might have discontinued their ART when they became more focused on spirituality and religion as coping mechanisms, but this could not be confirmed nor denied based on the available information. Ntshakala^17^ studied the QoL of ART patients in Swaziland and found that spirituality conflicted with ART adherence. Other researchers reported similar associations between spirituality and HRQoL.^8,17,18^

In our study, only 17.8% of respondents had coping experiences with overcoming previous difficult situations. Previous studies reported that PLWHA who experienced challenges in coping with ART had poorer ART adherence levels and poorer HRQoL mean scores compared to PLWHA without such challenges.^17,19^

The respondents of this study reported various reasons for defaulting ART. Most of the respondents were unemployed and even the employed ones had limited incomes. Lack of financial resources might have caused hardships to pay for transport to clinics to receive their monthly supplies of ARVs. The results of this study appear to be congruent with those of other studies identifying transportation costs, running out of pills and living far from clinics as reasons for defaulting ART.^20,21,22^

Similar to findings reported by other Botswana studies, respondents in this study reported depression as a reason for defaulting ART. Ehlers and Tshisuyi^23^ studied the factors that affected ART adherence among 300 ART patients in rural Botswana and found that depression was one of the top three reasons why respondents missed ARV doses. Lewis et al.^24^ reported that 48% of HIV-positive women (*n* = 62) in their Botswana study had been diagnosed with depression.

## Limitations

Of 362 ART defaulters identified from the participating clinics’ registers, only 128 (35.4%) could be contacted and comprised the accessible population for this study. As people in the rural areas of Botswana change locations frequently, most ART defaulters could not be traced at their previous addresses or phone numbers. The 24 ART defaulters who were contacted but refused to participate provided no reasons for their refusal. Consequently, no information could be obtained about ART defaulters who could not be contacted and those who refused to participate in the study. These inaccessible ART defaulters might have had different experiences than those who agreed to participate in the study.

Our study did not analyse the ART defaulters’ HRQoL scores over time, and thus possible time-dependent HRQoL changes were not identified.

The WHO HRQoL BREF instrument has not been tested extensively in Botswana for its cultural applicability. However, the respondents of this study did not encounter problems in replying to any item of this instrument.

## Conclusion

The low HRQoL mean scores in the social and environmental domains might signify inadequate family support, poor financial resources, poor physical living conditions and insecurity. The most common reason for ART defaulting was the inaccessibility of the clinics. Respondents were more likely to discontinue their treatment if ART clinics were far from their homes and if they felt better, ran out of ARVs, were depressed and did not have support. Addressing these issues could optimise the benefits of ART adherence and improve the HRQoL of PLWHA in Botswana. Further prospective studies using the same measures are required to confirm our study findings. Although all respondents discontinued ART for at least 3 months, their calculated HRQoL scores were moderate.

## References

[1] United Nations Joint Program on HIV and/or AIDS The gap report [homepage on the Internet]. Geneva; 2014 [cited 2015 May 04]. Available from: http://www.unaids.org/sites/default/files/media_asset/UNAIDS_Gap_report_en.pdf

[2] World Health Organization Quality of Life Group Introducing the WHOQOL Instruments. Department of Mental Health and Substance Dependency [homepage on the Internet]. Geneva: WHO; 1998 [cited 2011 Mar 16]; pp. 1, 2. Available from: http://depts.washington.edu/yqol/docs/WHOQOL_Info.pdf

[3] Tiwari MK, Verma S, Agrawal D, et al. Quality of life of patients with HIV infection. Indian J Soc Sci Res [serial online]. 2009 [cited 2013 Apr 15];6(2):79–86. Available from: http://ijssr.110mb.com/ijssr-oct-09/10-manoj.pdf

[4] Airoldi M, Zaccarelli M, Bisi L, et al. One-pill once-a-day HAART: A simplification strategy that improves adherence and quality of life of HIV-infected subjects. Patient Prefer Adherence [serial online]. 2010 [cited 2015 Apr 12];4:115–125. Available from: http://www.ncbi.nlm.nih.gov/pmc/articles/PMC2875721/pdf/ppa-4-115.pdf 10.2147/ppa.s10330 PMC2875721 20517472

[5] Liu C, Johnson L, Ostrow D, et al. Predictors for lower quality of life in the HAART era among HIV-infected men. J Acquir Immune Defic Syndr. 2006;42(4):470–477. 16810114 10.1097/01.qai.0000225730.79610.61

[6] Louwagie G, Bachmann M, Meyer K, et al. Highly active antiretroviral treatment and health related quality of life in South African adults with human immunodeficiency virus infection: A cross-sectional study. BioMedCentral Public Health [serial online]. 2007 [cited 2011 Feb 12];7:244 Available from: http://www.ncbi.nlm.nih.gov/pmc/articles/PMC2194770/pdf/1471-2458-7-244.pdf 10.1186/1471-2458-7-244 PMC2194770 17854510

[7] Ndubuka NO, Ehlers VJ Adult patients’ adherence to anti-retroviral treatment: A survey correlating pharmacy refill records and pill counts with immunological and virological indices. Int J Nurs Stud. 2011;48(11):1323–1329. 21570684 10.1016/j.ijnurstu.2011.04.006

[8] Tsevat J, Leonard AC, Szaflarski M, et al. Change in quality of life after being diagnosed with HIV: A multicenter longitudinal study. AIDS Patient Care STDS [serial online]. 2009 [cited 2014 Apr 20];23(11):931–937. Available from: http://www.ncbi.nlm.nih.gov/pmc/articles/PMC2832655/pdf/apc.2009.0026.pdf 10.1089/apc.2009.0026 PMC2832655 19821724

[9] Bisson GP, Stringer JSA Lost but not forgotten-the economics of improving patient retention treatment programs. PLoS Med [serial online]. 2009 [cited 2013 Jul 12];6:e1000174 Available from: http://www.plosmedicine.org/article/fetchObject.action?uri=info%3Adoi%2F10.1371%2Fjournal.pmed.1000174&representation=PDF 10.1371/journal.pmed.1000174 PMC2760758 19859529

[10] Chalker J, Andualem T, Minizi O, et al. Monitoring adherence and defaulting for antiretroviral therapy in 5 East African countries: An urgent need for standards. J Int Assoc Phys AIDS Care. 2008;7(4):193–199. 10.1177/1545109708320687 18626124

[11] Rachlis B, Ahmad F, van Lettow M, et al. Using concept mapping to explore why patients become lost to follow up from an antiretroviral therapy program in the Zomba District of Malawi. BioMedCentral Health Serv Res [serial online]. 2013 [cited 2013 Jun 30];13:210 Available from: http://www.biomedcentral.com/content/pdf/1472-6963-13-210.pdf 10.1186/1472-6963-13-210 PMC3698212 23758879

[12] Van Cutsem G, Ford N, Hildebrand K, et al. Correcting for mortality among patients lost to follow up on antiretroviral therapy in South Africa: A cohort analysis. *PLoS One* [serial online]. 2011 [cited 2013 Apr 13];6(2):e14684 Available from: http://www.plosone.org/article/info%3Adoi%2F10.1371%2Fjournal.pone.0014684 10.1371/journal.pone.0014684 PMC3040750 21379378

[13] Skevington SM, Norweg S, Standage M et al. Predicting quality of life for people living with HIV and AIDS: International evidence from seven cultures. AIDS Care. 2010;22(5):614–622. 20229378 10.1080/09540120903311466

[14] World Health Organization Quality of Life HIV Group WHOQOL-HIV Quality of Life assessment among people living with HIV and AIDS: Results from the field test. AIDS Care. 2004;16(7):882–889. 15385243 10.1080/09540120412331290194

[15] SAS Institute Inc Base SAS® 9.2 Procedure Guide. Cary, NC; 2009.

[16] Ndubuka NO Quality of life of ART patients in Botswana: Coping with challenges [unpublished D Lit et Phil thesis]. Pretoria: University of South Africa; 2015.

[17] Ntshakala TT Quality of life of people living with HIV and AIDS in Swaziland who are on antiretroviral therapy [unpublished D Lit et Phil (Health Studies) thesis]. Pretoria: University of South Africa; 2006.

[18] Fatiregun AA, Mofolorunsho KC, Osagbemi KG Quality of life of people living with HIV and/or AIDS in Kogi Sate, Nigeria. Benin J Postgrad Med [serial online]. 2009 [cited 2014 Feb 22];11(1):21–27. Available from: http://www.ajol.info/index.php/bjpm/article/view/48823/35172

[19] Mugavero M, Ostermann J, Whetten K, et al. Barriers to antiretroviral adherence: The importance of depression, abuse, and other traumatic events. AIDS Patient Care STDs. 2006;20(6):418–428. 16789855 10.1089/apc.2006.20.418

[20] Deribe K, Hailekiros F, Biadgilign S, et al. Defaulters from antiretroviral treatment in Jimma University Specialised Hospital, southwest Ethiopia. Trop Med Int Health [serial online]. 2008 [cited 2013 Jul 11];13(3):328–333. Available from: http://onlinelibrary.wiley.com/store/10.1111/j.1365-3156.2008.02006.x/asset/j.1365-3156.2008.02006.x.pdf?v=1&t=hj59bl9x&s=5c65d50c49fc362d1ac659516fb8f027b43bec38 10.1111/j.1365-3156.2008.02006.x 18298607

[21] McGuire M, Munyenembe T, Szumilin E, et al. Vital status of pre-ART and ART patients defaulting from care in rural Malawi. Trop Med Int Health. 2009;15(suppl. 1):55–62. 10.1111/j.1365-3156.2010.02504.x 20586961

[22] Sendagala S Factors affecting the adherence to antiretroviral therapy by HIV positive patients treated in a community based HIV/AIDS care programme in rural Uganda: A case of Tororo district [unpublished MPH (Health Studies) dissertation] Pretoria: University of South Africa; 2010.

[23] Ehlers VJ, Tshisuyi ET Adherence to antiretroviral therapy by adults in a rural area in Botswana. Curationis. 2015;38(1):1–8. 10.4102/curationis.v38i1.1255 PMC6091787 26244453

[24] Lewis EL, Mosepele M, Seloilwe E, et al. Depression in HIV-positive women in Gaborone, Botswana. Health Care Women Int [Serial online]. 2012 [cited 2014 Aug 30];33(4):375–386. Available from: http://www.tandfonline.com/doi/pdf/10.1080/07399332.2011.603871 10.1080/07399332.2011.603871 22420678

